# Lithium doped poly(3-hexylthiophene) for efficient hole transporter and sensitizer in metal free quaterthiophene dye treated hybrid solar cells

**DOI:** 10.1038/s41598-021-99762-3

**Published:** 2021-10-11

**Authors:** Arumugam Pirashanthan, Dhayalan Velauthapillai, Neil Robertson, Punniamoorthy Ravirajan

**Affiliations:** 1 Clean Energy Research Laboratory, Department of Physics, University of Jaffna, Jaffna, 40000 Sri Lanka; 2 Faculty of Engineering and Science, Western Norway University of Applied Sciences, 5020 Bergen, Norway; 3 School of Chemistry, University of Edinburgh, Joseph Black Building, Edinburgh, EH9 3FJ UK

**Keywords:** Energy harvesting, Solar cells, Materials science, Organic-inorganic nanostructures

## Abstract

This work focuses on the role of Lithium doped Poly(3-hexylthiophene)(P3HT) in metal-free quaterthiophene (4T) dye treated Titanium dioxide (TiO_2_) based hybrid solar cells. The dye treated hybrid solar cells with Lithium doped P3HT showed efficiencies (3.95%) of nearly a factor of four times higher than the pristine P3HT based control TiO_2_/4T/P3HT devices (1.04%). The enhancement of the efficiency is mainly due to highly efficient charge collection attributed to enhanced charge transport and light harvesting properties of Lithium doped P3HT polymer. The optimized solar cells with Lithium doped P3HT showed a high short circuit current density over 13 mA/cm^2^, under simulated irradiation of intensity 100 mW/cm^2^ with AM 1.5 filter. This significant increase in current density in TiO_2_/4T/doped P3HT solar cell is also confirmed by both the broadened External Quantum Efficiency spectrum and significant photoluminescence quenching upon replacement of pristine P3HT with doped P3HT on 4T dye treated TiO_2_ electrode. With Lithium doped Spiro-OMeTAD instead of Lithium doped P3HT, similar devices showed efficiencies over 3.30% under simulated irradiation of 100 mW/cm^2^ with AM 1.5 filter.

## Introduction

Hybrid solar cells with conjugated polymers as donors and metal oxide nanocrystals as acceptors have generated significant interest owing to their lightweight, low cost, mechanical flexibility, and simple solution processing methods^1–3^. These provide a simple model system to study the effects of interfacial properties and film morphology on the performance of bulk heterojunction solar cells^4^. Highly mesoporous structured Titanium dioxide (TiO_2_)^5,6^ and the relatively stable^7,8^ simple homopolymer poly(3-hexylthiophene)(P3HT) are some of the most extensively used materials in the field of solar cells research. However, these hybrid TiO_2_/P3HT solar cells have a limited power conversion efficiency (PCE) due to several reasons, including the narrow spectral response of the polymer, poor chemical compatibility and poor quality of the interface between inorganic acceptor Titanium dioxide and organic polymer donor^9,10^. Several studies have been carried in past decades to overcome above limitations^11^. The reverse bias annealing/UV exposure procedure reorients defects and dangling bonds at the metal oxide—polymer interface as evidenced in Pandey et al. and Ravirajan et al.^12,13^.The interface modifier between metal oxide nanoparticles and the polymer helps to improve the carrier generation, charge collection and transport of carriers in these hybrid solar cells^14,15^. A range of novel organic and inorganic materials such as self-assembled monolayers^16–18^, carbonaceous materials^19^ and inorganic inter layers^20,21^ have been employed as interface modifiers which results in improved power conversion efficiencies of the hybrid solar cells. Further, a variety of dyes has been applied as interface modifiers^22–27^. Crucial light absorption in the visible region from the solar spectrum has been observed, due to the high charge transfer by metal complex dyes^28^. A broad absorption spectrum, optimal excited and ground state energy levels, relatively long excited-state lifetime and good (electro) chemical stability induce the best photovoltaic performance in the solar cells when Ru complexes are used as interface modifiers^29^. It is found that these dyes extend the spectral response by participating in exciton creation and reducing the carrier recombination at the TiO_2_/P3HT interface, which results in an improved short circuit current density ($J_{SC}$) and open-circuit voltage ($V_{OC}$) in order to enhance the overall performance^14,22^. However, the extension of spectral response of P3HT towards either visible or UV-region depends on the absorption range of the dye used at the TiO_2_/P3HT interface^14^ and it was proposed that UV exposure modifies the nature or density of surface trapping species in the nanocrystalline TiO_2_, resulting in reduced recombination rates and a higher efficiency of collection of photogenerated charges^13^.

It was found that the insertion of quaterthiophene based 4T dye at Titanium dioxide/Poly (3-hexylthiophene) interface showed an enhanced efficiency^22,30^ and, improved hole mobility of the polymer nanocomposite by up to two orders of magnitude compared to the corresponding control^31^. This electron-rich thiophene cyanoacrylic acid group containing metal-free dye led to devices with high $V_{OC}$ via generation of dipole moments at the interface^30,32,33^. Molar extinction coefficient (*ε*) of 4T dye is also higher compared to commercial ruthenium based N719 and Z907 dyes^22^. The stability and carrier generation is highly influenced by the ε of light absorbers^34^. Moreover, the stability and performance reproducibility of a solar cell is highly influenced by additive materials that are used for the fabrication. The Bis(trifluoromethane)sulfonimide lithium salt (LiTFSI) and 4-tert-butylpyridine (tBP) are the most common additives for Spiro-OMeTAD hole transporter in dye sensitised and perovskite solar cells. These additives help to overcome issues such as high series resistances and poor photovoltaic performances of undoped Spiro-OMeTAD^35^. Further, it was found that the hole mobility of Spiro-OMeTAD hole transporter is highly influenced by photodoping of Spiro-OMeTAD with oxygen, facilitated by the presence of LiTFSI^36–38^. The Coulombic attraction of the bound charge carrier pairs at the TiO_2_/polymer interface leads to carrier recombination^13,22,39^. Subsequently, lithium ions help to reduce the recombination by compensating the exited free electrons in the conduction band (CB) of TiO_2_^38^. In addition to lithium ions, tBP controls the carrier recombination through adsorption onto the dyed mesoporous titanium dioxide in places not covered by dye molecules. Furthermore, tBP induces a TiO_2_ CB upshift due to its molecular dipole moment. The energy-level difference between the quasi-Fermi level of polymer hole transporter and the mesoporous metal oxide determines the $V_{OC}$ of the particular nanocomposite based solar cell. Shifting the conduction band (CB) edge away from the lowest unoccupied molecular orbital (LUMO) level of polymer hole transporting material (HTM) can improve the $V_{OC}$^32,40,41^. The tBP-LiTFSI doped Spiro-OMeTAD is a widely used HTM in solid-state dye sensitised solar cells and Perovskite solar cells^42,43^. Pristine P3HT is a well-known hole transporter and a good absorber as reported in the field of hybrid titanium dioxide based solar cells^44,45^. There are only a few reports on utilising tBP-LiTFSI doped P3HT as a hole transporter, notably in Perovskite solar cells^46^. To the best of our knowledge, this is the first successful report on utilising tBP-LiTFSI doped P3HT as an HTM in hybrid Titanium dioxide/Poly(3-hexylthiophene) solar cells.

**Figure 1:**
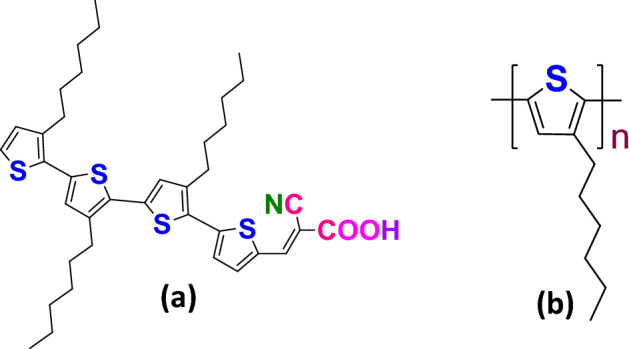
Chemical structures of (**a**) 4T dye and (**b**) P3HT polymer. Both structures have hexyl-substituted thiophene ring as a common unit.

This work focuses on studying the role of bis(trifluoromethane) sulfonimide lithium salt (LiTFSI) and 4-tert-butylpyridine (tBP) doped Poly(3-hexylthiophene) (P3HT) in Titania based hybrid solar cells with a metal free quaterthiophene cyanoacrylic acid group ((E)-2-cyano-3-(3′,3′′,3′′′-trihexyl-[2,2′:5′,2′′:5′′,2′′′-quaterthiophene]-5-yl) acrylicacid)(4T) dye as an interface modifier. Figure 1 shows the chemical structures of both 4T dye and P3HT polymer.

The tail-like thiophene based structure of 4T can easily penetrate through the mesoporous TiO_2_, thus the TiO_2_/P3HT interface is enhanced via facilitating higher active surface area between TiO_2_ and P3HT. Photovoltaic parameters of solar cells with the dye treated TiO_2_ electrode and doped Spiro-OMeTAD were also compared. Here, Bis(trifluoromethane)sulfonimide lithium salt (LiTFSI) and 4-tert-butylpyridine (tBP) are used as common additives for the doped hole transporters. This confirmed our previous studies that the insertion of dye at the TiO_2_/P3HT interface improves the efficiency. However, TiO_2_/4T/doped P3HT devices significantly improved with a champion efficiency of 3.95% by exhibiting a high $J_{SC}$ of about 13 mA/cm^2^ which is consistent with the higher hole mobility value of the P3HT reported in TiO_2_/P3HT nanocomposite with 4T dye^31^ and broader spectral absorption of the doped P3HT.

## Results and discussion

**Figure 2:**
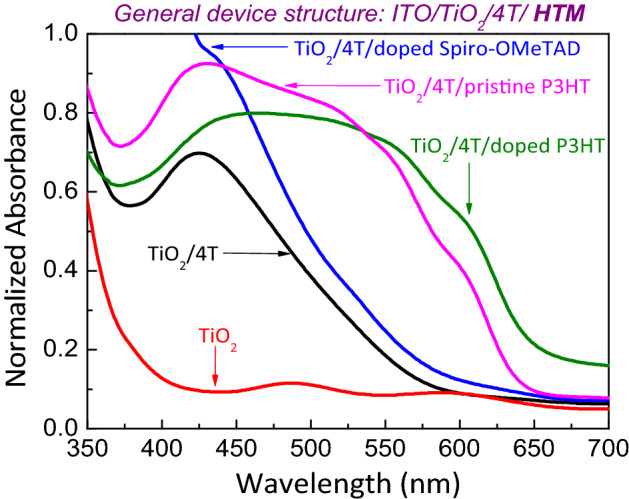
optical absorption spectra of 4T dye treated mesoporous TiO_2_ electrode and doped P3HT or doped Spiro-OMeTAD along with the control TiO_2_/4T/P3HT structure and porous TiO_2_ electrode.

Figure 2 shows the optical absorption spectra of 4T dye treated mesoporous TiO_2_ electrode and doped P3HT/doped Spiro-OMeTAD along with the control TiO_2_/4T/P3HT structure and porous TiO_2_ electrode. The figure reveals that the 4T dye treated mesoporous films of 600 nm exhibit very high absorption which is due to higher Molar Extinction Coefficient (ε) as reported in refs^22,30^. Further, the comparison of absorption of pristine P3HT, doped P3HT and doped Spiro-OMeTAD HTM coated TiO_2_ films shows a broad optical spectral response in the presence of 4T interface modifier and doped P3HT.

**Figure 3:**
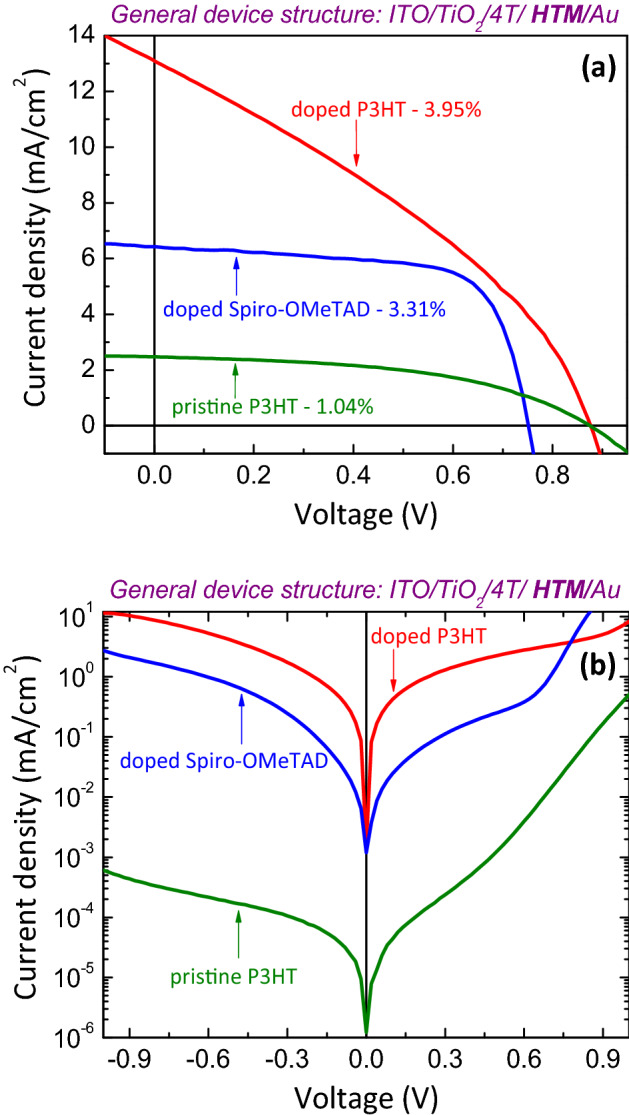
(**a**) J–V characteristics of ITO/TiO_2_/4T/HTM/Au solar cells under simulated irradiation of 100 mW/cm^2^ (1 sun) with Air Mass 1.5 filter and (**b**) semi-log J–V plot of the solar cells in dark. Here, the 4T dye is used as an interface modifier and HTMs are pristine P3HT, doped P3HT and doped Spiro-OMeTAD.

**Figure 4:**
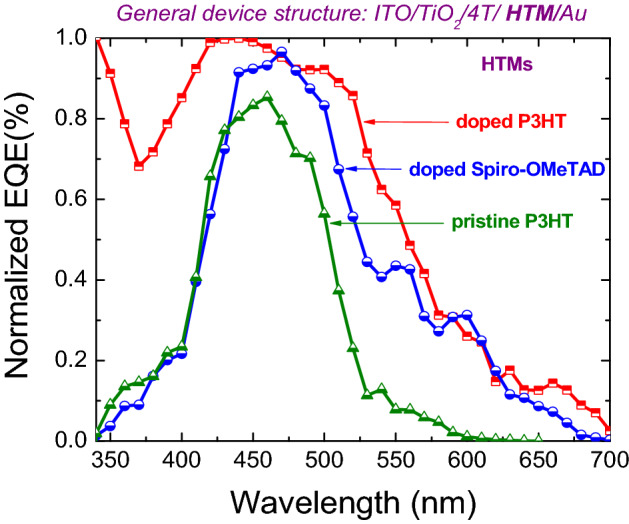
External quantum efficiency (EQE) of ITO/TiO_2_/4T/HTM/Au solar cells. Here, the 4T dye is used as an interface modifier and HTMs are pristine P3HT, doped P3HT and doped Spiro-OMeTAD.

The figure further compares the spectral response of doped P3HT and doped Spiro-OMeTAD HTM layers. Spectral response of 4T dye treated mesoporous TiO_2_ electrode with doped P3HT is broader than that of with doped Spiro-OMeTAD. Further Fig. 2, shows that the doped P3HT with the presence of 4T dye showed a broadened and red-shifted spectrum compared to the pristine P3HT with 4T dye. This may be the reason behind the higher $J_{SC}$ found in 4T dye treated mesoporous TiO_2_ electrode with doped P3HT solar cells as shown in the Fig. 3. Higher $J_{SC}$ is consistent with the corresponding EQE spectrum of device shown in Fig. 4. This figure clearly shows the increased carrier generation/collection in the visible region for the device with doped P3HT when compared to the other HTMs, pristine P3HT and doped Spiro-OMeTAD.

External quantum efficiency (EQE) measurement was carried out with optimized hybrid solar cells with the structures of ITO/TiO_2_/4T/pristine P3HT/Au, ITO/TiO_2_/4T/doped P3HT/Au and ITO/TiO_2_/4T/doped Spiro-OMeTAD/Au. In Fig. 4, the EQE spectrum of TiO_2_/4T/doped P3HT/Au device is broadened compared to other devices, which is attributed to the influence of the red-shifted wider absorption of TiO_2_/4T/doped P3HT nanocomposite in Fig. 1. Moreover, the EQE spectrum of TiO_2_/4T/doped P3HT/Au increased to over 85% near the peak absorption of 4T around 426 nm. This shows the dominant role of 4T dye in carrier generation, however it was enhanced by doped P3HT when it combines with 4T in order to form 4T/doped P3HT nanocomposite. This better performance of the device with 4T and doped P3HT could be due to the better compatibility of the quaterthiophene based 4T dye with the Poly(3-hexyl thiophene) polymer as both have common thiophene units in their structure.

Figure 3a further shows that the insertion of 4T dye at the TiO_2_/P3HT and TiO_2_/doped P3HT interface resulted in a high open-circuit voltage around 0.87 V which is consistent with our previous work^31^. This is probably due to the increased number of electron-rich thiophene units present with 4T dye treated TiO_2_/P3HT and TiO_2_/doped P3HT nanocomposites, which leads to a dipole moment at the interface^30,33,47^. Furthermore, the current density of 4T dye treated devices with both doped Spiro-OMeTAD and doped P3HT were around 6.43 mA/cm^2^ and 13.02 mA/cm^2^, respectively. The experimental values were verified by integrating the EQE spectrum of the corresponding devices. The resulting $J_{SC}$ is five times higher with doped P3HT than the corresponding control device with pristine P3HT. The dark J-V in Fig. 3b shows that series resistance of the device with doped P3HT decreases significantly due to increased hole-mobility of doped P3HT as a result of the presence of Lithium salt as a dopant^48,49^. Further, significantly increased dark current (order of four) of the doped P3HT device in comparison with pristine P3HT device shows an increased number of carriers participating in electron–hole pair generation. The overall efficiency (ɳ) of doped P3HT device is 3.95% with enhanced $J_{SC}$ and higher $V_{OC}$ values, whereas the corresponding control pristine P3HT solar cell exhibited an efficiency of 1.04%.

The 4T dye treated hybrid solar cells fabricated with pristine P3HT and doped P3HT HTMs were next compared with tBP-LiTFSI doped Spiro-OMeTAD HTM. The doped Spiro-OMeTAD and doped P3HT solar cells showed champion efficiencies around 3.31% and 3.95%, respectively (Table 1). This enhanced efficiency in TiO_2_/4T/doped P3HT/Au device is probably attributed to broader spectral response of TiO_2_/4T/doped P3HT and the increased hole-mobility of doped P3HT due to the presence of Lithium salt as a dopant. Furthermore, it has been reported that Lithium salt can increase of hole mobility of polymer^48,49^.

**Table 1:** experimental J-V parameters of champion devices with three different HTMs.

HTM	$J_{SC} \left( {{\text{mA/cm}}^{2} } \right)$	$V_{OC} \left( {\text{V}} \right)$	$FF$	ɳ%	$R_{s} \left( \Omega \right)$	$R_{sh} \left( {{\text{k}}\Omega } \right)$
Pristine P3HT	2.47	0.87	0.48	1.04	2,332	44.25
doped Spiro-OMeTAD	6.43	0.75	0.68	3.31	248	27.79
doped P3HT	13.02	0.87	0.34	3.95	573	2.54

**Figure 5:**
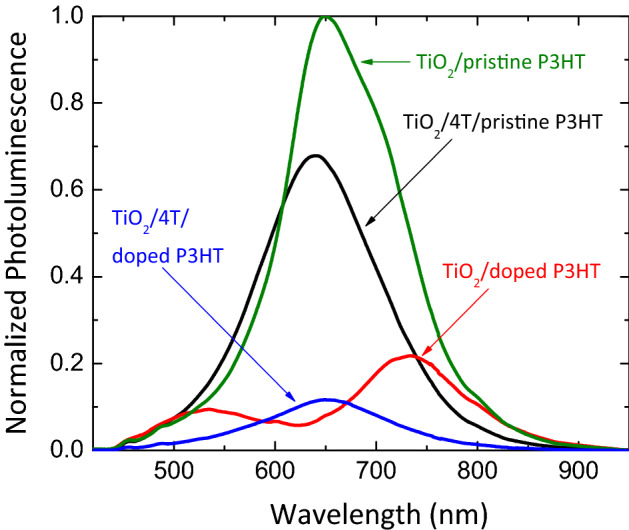
Normalized Photoluminescence (PL) spectra of TiO_2_/P3HT, TiO_2_/4T/P3HT, TiO_2_/doped P3HT and TiO_2_/4T/doped P3HT films. Here, the interface of TiO_2_/P3HT, TiO_2_/doped Spiro and TiO_2_/doped P3HT were modified with 4T dye.

The role of 4T dye and Lithium dopants were studied with photoluminescence analysis. Figure 5 shows the normalized photoluminescence spectra of the films recorded by exciting the films with a 405 nm solid-state laser at 300 K. This wavelength was used to eliminate the effect of deep UV laser (325 nm) on organic materials and laser excitation close to the bandgap of the semiconducting material. Further, Fig. 5 shows that the photoluminescence of TiO_2_/polymer nanocomposite is significantly quenched upon the interface modification with 4T dye. The figure further shows that the PL emission is further quenched in the presence of doped P3HT^50^. This significant quenching of TiO_2_/4T/doped P3HT emission indicates the enriched exciton dissociation via reduced carrier recombination at the interface of TiO_2_ nanocrystals and doped P3HT due to the enhanced hole mobility of P3HT in the presence of Li-TFSI content, and as well as increased electron-rich thiophene rings in the TiO_2_/4T/doped P3HT nanocomposite.

## Conclusions

This study concludes that the efficiency of the hybrid TiO_2_/polymer solar cells can significantly be increased by doping the hole transporters (P3HT, Spiro-OMeTAD) with LiTFSI and tBP and treating the TiO_2_ electrodes with a metal free quaterthiophene 4T dye. The optimised solar cells showed a high short circuit current density ($J_{SC}$) of 13 mA/cm^2^ with an enhanced efficiency of 3.95% which is nearly a factor of four times higher than the efficiency of the corresponding control device under 1 sun illumination with an AM 1.5 filter. The dark J–V, Photoluminescence quenching and EQE data confirms that enhanced performance is due to improved hole transportation of doped P3HT HTM and the improved TiO_2_/P3HT interface by 4T dye. The largest remaining limitation of the optimised cell arises from the low shunt resistance and resulting low fill factor of 0.34. Future work will include a focus on this aspect such that the PCE can be further enhanced.

## Methods

### Fabrication of hybrid solar cells

The solar cells were fabricated as reported elsewhere^14,22^. Cleaned Indium Tin Oxide (ITO) coated glass substrates were coated with aerosol spray pyrolysis deposition of diluted solution of Titanium (iv) isopropoxide (98%) and acetylacetone (99%) precursor in ethanol at a substrate temperature of 400 °C and, sintered at 500 °C for 30 min in order to form ~ 50 nm thick compact TiO_2_ layer^19^. Thereafter, a dissolved solution (240 mg/mL) of Dyesol 18NRT TiO_2_ paste in tetrahydrofuran (0.005% H_2_O) was spin coated on the top of the compact TiO_2_ and allowed to sinter at 450 °C for 30 min to form a mesoporous TiO_2_ layer. The thickness of the mesoporous TiO_2_ layer was maintained at 600 nm for all the tested devices to maintain uniformity^22^. Once cooled, these mesoporous films were modified with 4T (Mw = 678.05) dye at 0.3 mM concentration by dip coating^31^. The dye solutions were prepared using the 1:1 solvent mixture of acetonitrile (99.8%) and tert-butanol (99.5%). After dye dipping, the electrodes were rinsed in the same solvent to remove the excess dye in the nanoporous TiO_2_ layer.

The dyed electrodes were used directly for the fabrication of the solar cell with three different hole transporting layers such as pristine P3HT, doped P3HT and doped Spiro OMeTAD. The pristine P3HT layer was spin coated with 25 mg/ml concentrated solution of P3HT in chlorobenzene (99.8%) at 2000 rpm for 30 s. For the doped Spiro-OMeTAD solution, first 1 mL of 72.3 mg/mL concentrated solution of Spiro-OMeTAD (99%) in chlorobenzene, 17.5 μL of 520 mg/mL concentrated solution of bis(trifluoromethane) sulfonimide lithium salt (LiTFSI) (96%) in Acetonitrile and 28.8 μL of 4-tert-butylpyridine (tBP) (96%) were added together and allowed to mix well for 40 minutes^51^. Once the clear solution was observed, the doped Spiro-OMeTAD solution was dispensed on the substrate and allowed to spread across the total area of the substrate through the spin coating. For the doped P3HT solution, first 1 mL of 25 mg/mL concentrated solution of P3HT in chlorobenzene, 12 μL of 520 mg/mL concentrated solution of bis(trifluoromethane)sulfonimide lithium salt (LiTFSI) in Acetonitrile, and 11.4 μL of 4-tert-butylpyridine (tBP) were added together and allowed to mix well^46^. Preheated doped P3HT solution was deposited on top of the dye-modified TiO_2_ electrodes via spin coating.

Finally, all the HTM coated films were stored in the dark overnight prior to the deposition of thermally evaporated 80 nm thick gold (Au 99.8%) electrode under high vacuum. After the Au deposition, a conductive silver paste (107.87 g/mol) was added on top of each Au electrode, followed by annealing the device at 120 °C under a nitrogen environment to provide a better electrical contact between the fabricated solar cells and the sample holder.

### Optical and electrical characterization

Optical absorbance spectra of the TiO_2_/dye and TiO_2_/dye/HTM layered films were recorded by using a JENWAY 6800 UV/Vis. Spectrophotometer, which was controlled using Flight Deck software. Photoluminescence spectra were recorded with Horiba Jobin Yvon iHR320 spectrometer which is equipped with UV–Vis and NIR Photomultiplier tubes. Current–voltage characterization of fabricated solar cells was tested, and the curves were recorded with a computer-controlled Keithley 2400 source meter unit under illuminations of intensity of 100 mW/cm^2^ (1 sun) provided by a solar simulator (Peccell) with AM (Air Mass) 1.5 spectral filter. The External quantum efficiency measurements were carried out using a Monochromator (Newport) and a calibrated silicon photodiode (818 UV).

## References

[1] Ravirajan P, Haque SA, Durrant JR, Bradley DDC, Nelson J (2005). The effect of polymer optoelectronic properties on the performance of multilayer hybrid polymer/TiO_2_ solar cells. Adv. Funct. Mater..

[2] Ishwara T (2008). Influence of polymer ionization potential on the open-circuit voltage of hybrid polymer/TiO_2_ solar cells. Appl. Phys. Lett..

[3] Wu F (2017). Balanced dipole effects on interfacial engineering for polymer/TiO_2_ array hybrid solar cells. Nanoscale Res. Lett..

[4] Grupp A (2017). Incoherent pathways of charge separation in organic and hybrid solar cells. J. Phys. Chem. Lett..

[5] Giordano F (2016). Enhanced electronic properties in mesoporous TiO_2_ via lithium doping for high-efficiency perovskite solar cells. Nat. Commun..

[6] Kajana T (2020). Structural and photoelectrochemical characterization of heterostructured carbon sheet/Ag_2_MoO_4_-SnS/Pt photocapacitor. J. Photochem. Photobiol. A Chem..

[7] Holliday S (2016). High-efficiency and air-stable P3HT-based polymer solar cells with a new non-fullerene acceptor. Nat. Commun..

[8] Shanmugaratnam S, Velauthapillai D, Ravirajan P, Christy AA, Shivatharsiny Y (2019). CoS_2_/TiO_2_ nanocomposites for hydrogen production under UV irradiation. Materials (Basel).

[9] Ravirajan P (2006). Hybrid polymer/zinc oxide photovoltaic devices with vertically oriented ZnO nanorods and an amphiphilic molecular interface layer. J. Phys. Chem..

[10] Pei J (2016). optimizing the performance of tio_2_/p3ht hybrid solar cell by effective interfacial modification. Chem. Phys. Lett..

[11] Bouclé J, Ravirajan P, Nelson J (2007). Hybrid polymer–metal oxide thin films for photovoltaic applications. J. Mater. Chem..

[12] Pandey AK (2007). Reverse biased annealing: Effective post treatment tool for polymer/nano-composite solar cells. Org. Electron..

[13] Ravirajan P, Atienzar P, Nelson J (2012). Post-processing treatments in hybrid polymer/titanium dioxide multilayer solar cells. J. Nanoelectron. Optoelectron..

[14] Pirashanthan A (2020). A multifunctional ruthenium based dye for hybrid nanocrystalline titanium dioxide/poly(3-hexylthiophene) solar cells. Mater. Lett..

[15] Thu C (2018). Role of the metal-oxide work function on photocurrent generation in hybrid solar cells. Sci. Rep..

[16] Loheeswaran S, Thanihaichelvan M, Ravirajan P, Nelson J (2017). Controlling recombination kinetics of hybrid poly-3-hexylthiophene (P3HT)/titanium dioxide solar cells by self-assembled monolayers. J. Mater. Sci. Mater. Electron..

[17] Kırbıyık Ç (2019). Improving the performance of inverted polymer solar cells through modification of compact TiO_2_ layer by different boronic acid functionalized self-assembled monolayers. Appl. Surf. Sci..

[18] Pei J (2018). Influence of organic interface modification layer on the photoelectric properties of ZnO-based hybrid solar cells. J. Photochem. Photobiol. A Chem..

[19] Balashangar K (2018). Multi-walled carbon nanotube incorporated nanoporous titanium dioxide electrodes for hybrid polymer solar cells. Mater. Lett..

[20] Armstrong CL (2015). Influence of an inorganic interlayer on exciton separation in hybrid solar cells. ACS Nano.

[21] Loheeswaran S, Balashangar K, Jevirshan J, Ravirajan P (2014). Controlling recombination kinetics of hybrid nanocrystalline titanium dioxide/polymer solar cells by inserting an alumina layer at the interface. J. Nanoelectron. Optoelectron..

[22] Pirashanthan A, Murugathas T, Robertson N, Ravirajan P, Velauthapillai D (2019). A quarterthiophene-based dye as an efficient interface modifier for hybrid titanium dioxide/poly(3-hexylthiophene)(P3HT) solar cells. Polymers (Basel).

[23] Mecking S (2019). Tailored interface energetics for efficient charge separation in metal oxide-polymer solar cells. Sci. Rep..

[24] Calloni A (2014). Photoemission study of the poly(3-hexylthiophene)/TiO_2_ interface and the role of 4-mercaptopyridine. Thin Solid Films.

[25] Rajaramanan T, Natarajan M, Ravirajan P, Senthilnanthanan M, Velauthapillai D (2020). Ruthenium (Ru) doped titanium dioxide (P25) electrode for dye sensitized solar cells. Energies.

[26] Moon SJ (2011). Enhanced light harvesting in mesoporous TiO_2_/P3HT hybrid solar cells using a porphyrin dye. Chem. Commun..

[27] Yan W, Jiang D, Liu Q, Kang Q, Zhou F (2019). Solar cells constructed with polythiophene thin films grown along tethered thiophene-dye conjugates via photoelectrochemical polymerization. ACS Appl. Mater. Interfaces.

[28] Rasalingam S, Peng R, Wu CM, Mariappan K, Koodali RT (2015). Robust and effective Ru-bipyridyl dye sensitized Ti-MCM-48 cubic mesoporous materials for photocatalytic hydrogen evolution under visible light illumination. Catal. Commun..

[29] Pirashanthan A (2021). Synthesis of a carboxylic acid-based ruthenium sensitizer and its applicability towards dye-sensitized solar cells. Sol. Energy.

[30] Planells M, Abate A, Snaith HJ, Robertson N (2014). Oligothiophene interlayer effect on photocurrent generation for hybrid TiO_2_/P3HT Solar Cells. ACS Appl. Mater. Interfaces.

[31] Prashanthan K (2017). Enhancement of hole mobility in hybrid titanium dioxide/poly(3-hexylthiophene) nanocomposites by employing an oligothiophene dye as an interface modifier. J. Mater. Chem. C.

[32] Malloci G, Binda M, Petrozza A, Mattoni A (2013). Role of molecular thermodynamical processes at functionalized polymer/metaloxide interfaces for photovoltaics. J. Phys. Chem. C.

[33] Reeja-Jayan B (2015). Oligomeric interface modifiers in hybrid polymer solar cell prototypes investigated by fluorescence voltage spectroscopy. Phys. Chem. Chem. Phys..

[34] Dissanayake MAKL, Jaseetharan T, Senadeera GKR, Thotawatthage CA (2018). A novel, PbS: Hg quantum dot-sensitized, highly efficient solar cell structure with triple layered TiO_2_ photoanode. Electrochim. Acta.

[35] Fan WJ (2020). Improving the fill factor of perovskite solar cells by employing an amine-tethered diketopyrrolopyrrole-based polymer as the dopant-free hole transport layer. ACS Appl. Energy Mater..

[36] Cappel UB, Daeneke T, Bach U (2012). Oxygen-induced doping of spiro-MeOTAD in solid-state dye-sensitized solar cells and its impact on device performance. Nano Lett..

[37] Snaith HJ, Grätzel M (2006). Enhanced charge mobility in a molecular hole transporter via addition of redox inactive ionic dopant: Implication to dye-sensitized solar cells. Appl. Phys. Lett..

[38] Yang L (2018). Experimental and theoretical investigation of the function of 4-tert-butyl pyridine for interface energy level adjustment in efficient solid-state dye-sensitized solar cells. ACS Appl. Mater. Interfaces.

[39] Armstrong CL (2015). Influence of an inorganic inter layer on exciton separation in hybrid solar cells. ACS Nano.

[40] Habisreutinger SN, Noel NK, Snaith HJ, Nicholas RJ (2017). Investigating the role of 4-tert butylpyridine in perovskite solar cells. Adv. Energy Mater..

[41] Weickert J (2013). Synergistic effects of interfacial modifiers enhance current and voltage in hybrid solar cells. APL Mater..

[42] Uthayaraj S (2019). Powder pressed cuprous iodide (CuI) as a hole transporting material for perovskite solar cells. Materials (Basel).

[43] Hawash Z, Ono LK, Qi Y (2018). Recent advances in spiro-MeOTAD Hole transport material and its applications in organic-inorganic halide perovskite solar cells. Adv. Mater. Interfaces.

[44] Liao W, Wu J (2013). Efficient electron collection in hybrid polymer solar cells: In-situ-generated ZnO/poly(3-hexylthiophene) scaffolded by a TiO_2_ nanorod array. J. Phys. Chem..

[45] Wang D, Tao H, Zhao X, Zhang T, Han J (2015). TiO_2_/P3HT hybrid solar cell with efficient interface modification by organic and inorganic materials: A comparative study. J. Nanosci. Nanotechnol..

[46] Nia NY, Matteocci F, Cina L, Di Carlo A (2017). High-efficiency perovskite solar cell based on poly(3-hexylthiophene): Influence of molecular weight and mesoscopic scaffold layer. Chemsuschem.

[47] Ben Dkhil S (2017). P-type semiconductor surfactant modified zinc oxide nanorods for hybrid bulk heterojunction solar cells. Sol. Energy Mater. Sol. Cells.

[48] Kim SK (2020). Recyclable high-performance polymer electrolyte based on a modified methyl cellulose-lithium trifluoromethanesulfonate salt composite for sustainable energy systems. Chemsuschem.

[49] Abate A (2013). Lithium salts as ‘redox active’ p-type dopants for organic semiconductors and their impact in solid-state dye-sensitized solar cells. Phys. Chem. Chem. Phys..

[50] Venkatachalapathy V, Galeckas A, Kuznetsov AY (2014). Tunneling in ZnO/ZnCdO quantum wells towards next generation photovoltaic cells. Sol. Energy.

[51] Zhao Q (2019). High efficiency perovskite quantum dot solar cells with charge separating heterostructure. Nat. Commun..

